# Fuzzy-Logic Based Detection and Characterization of Junctions and Terminations in Fluorescence Microscopy Images of Neurons

**DOI:** 10.1007/s12021-015-9287-0

**Published:** 2015-12-23

**Authors:** Miroslav Radojević, Ihor Smal, Erik Meijering

**Affiliations:** Biomedical Imaging Group Rotterdam, Departments of Medical Informatics and Radiology, Erasmus University Medical Center, Rotterdam, the Netherlands

**Keywords:** Neuron reconstruction, Junction detection, Bifurcation detection, Termination detection, Fuzzy logic, Fluorescence microscopy, Image analysis

## Abstract

Digital reconstruction of neuronal cell morphology is an important step toward understanding the functionality of neuronal networks. Neurons are tree-like structures whose description depends critically on the junctions and terminations, collectively called critical points, making the correct localization and identification of these points a crucial task in the reconstruction process. Here we present a fully automatic method for the integrated detection and characterization of both types of critical points in fluorescence microscopy images of neurons. In view of the majority of our current studies, which are based on cultured neurons, we describe and evaluate the method for application to two-dimensional (2D) images. The method relies on directional filtering and angular profile analysis to extract essential features about the main streamlines at any location in an image, and employs fuzzy logic with carefully designed rules to reason about the feature values in order to make well-informed decisions about the presence of a critical point and its type. Experiments on simulated as well as real images of neurons demonstrate the detection performance of our method. A comparison with the output of two existing neuron reconstruction methods reveals that our method achieves substantially higher detection rates and could provide beneficial information to the reconstruction process.

## Introduction

The complexity and functionality of the brain depend critically on the morphology and related interconnectivity of its neuronal cells (Kandel et al. 2000; Ascoli 2002; Donohue and Ascoli 2008). To understand how a healthy brain processes information and how this capacity is negatively affected by psychiatric and neurodegenerative diseases (Anderton et al. 1998; Lin and Koleske 2010; Ṡiṡková et al. 2014) it is therefore very important to study neuronal cell morphology. Advanced microscopy imaging techniques allow high-sensitivity visualization of individual neurons and produce vast amounts of image data, which are shifting the bottleneck in neuroscience from the imaging to the data processing (Svoboda 2011; Peng et al. 2011; Senft 2011; Halavi et al. 2012) and call for a high level of automation. The first processing step toward high-throughput quantitative morphological analysis of neurons is their digital reconstruction from the image data. Many methods have been developed for this in the past decades (Meijering 2010; Donohue and Ascoli 2011) but the consensus of recent studies is that there is still much room for improvement in both accuracy and computational efficiency (Liu 2011; Svoboda 2011).

**Fig. 1:**
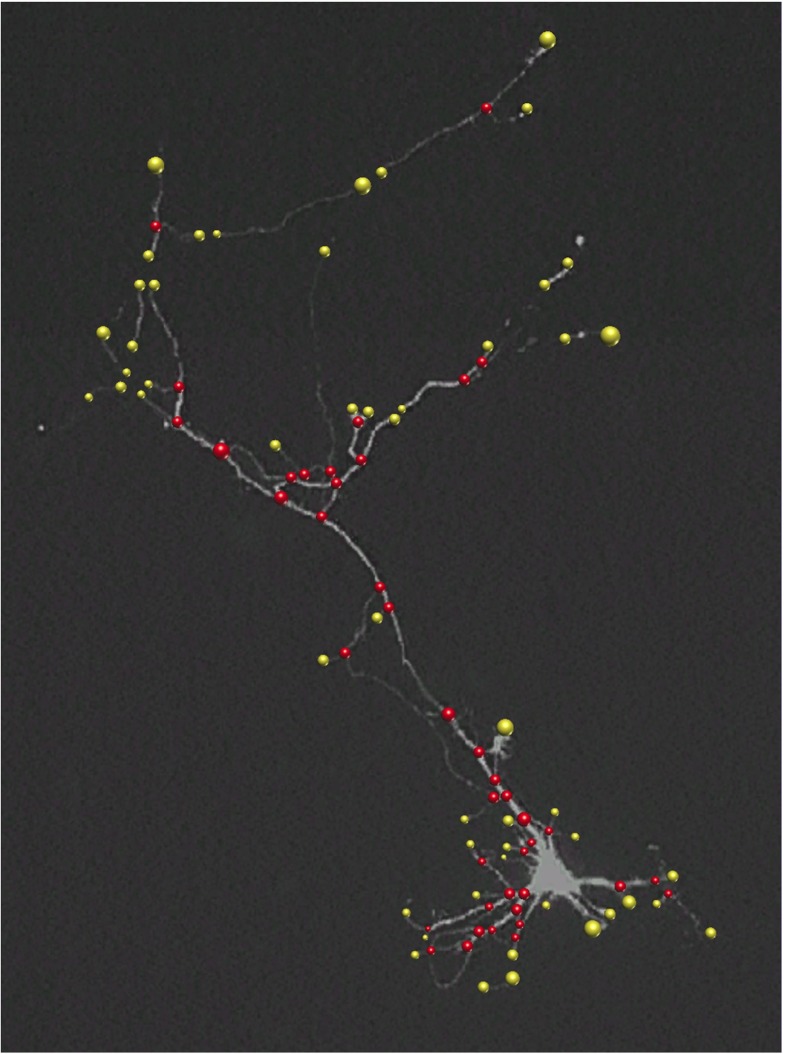
Fluorescence microscopy image of a neuron with manually indicated junctions (*red circles*) and terminations (*yellow circles*). The radius of each annotated critical-point region reflects the size of the underlying image structure

A key aspect of any neuron reconstruction method is the detection and localization of terminations and junctions of the dendritic (and axonal) tree, collectively called “critical points” in this paper (Fig. 1), which ultimately determine the topology and faithfulness of the resulting digital representation. Roughly there are two ways to extract these critical points in neuron reconstruction (Al-Kofahi et al. 2008; Meijering 2010; Basu et al. 2013). The most often used approach is to start with segmentation or tracing of the elongated image structures and then to infer the critical points, either afterwards or along the way, by searching for attachments and endings in the resulting subsets (Dima et al. 2002, Xiong et al. 2006, Narro et al. 2007, Vasilkoski and Stepanyants 2009, Bas and Erdogmus 2011, Chothani et al. 2011, Dehmelt et al. 2011, Ho et al. 2011, Choromanska et al. 2012, Xiao and Peng 2013). This approach depends critically on the accuracy of the initial segmentation or tracing procedure, which usually is not designed to reliably capture critical points in the first place and thus often produces very fragmented results, requiring manual postprocessing to fix issues (Peng et al. 2011; Luisi et al. 2011; Dercksen et al. 2014). The reverse approach is to first identify critical points in the images and then to use these as seed points for tracing the elongated image structures. Critical points can be obtained either by manual pinpointing, as in semiautomatic tracing methods (Meijering et al. 2004, Schmitt et al. 2004, Narro et al. 2007, Lu et al. 2009, Peng et al. 2010, Longair et al. 2011), or by fully automatic detection using sophisticated image filtering and pattern recognition methods (discussed in the next section). The latter approach has barely been explored for neuron reconstruction, but if reliable detectors can be designed, they provide highly valuable information to the reconstruction process.

Here we present a novel method – which we coin Neuron Pinpointer (NP) – for fully automatic detection and characterisation of critical points in fluorescence microscopy images of neurons. We describe and evaluate the method for studies where single (cultured) neurons are imaged in 2D although all aspects of the method can in principle be extended to 3D. The method may also be useful for reconstruction approaches based on 2D projections (Zhou et al. 2015). For computational efficiency the method starts with an initial data reduction step, based on local variation analysis, by which obvious background image regions are excluded. In the remaining set of foreground regions the method then explores the local neighborhood of each image pixel and calculates the response to a set of directional filters. Next, an iterative optimization scheme is used for robust peak selection in the resulting angular profile, and a set of corresponding features relevant for the detection task is computed. The feature set is then further processed to make a nonlinear decision on the presence of a critical point and its type (termination or junction) at each foreground image pixel. To conveniently deal with ambiguity and uncertainty in the data, the decision-making is carried out by a fuzzy-logic rule-based system using predefined rules specifically designed for this task. The presented work aims to facilitate the task of automatic neuron reconstruction by contributing a general scheme for extracting critical points that can serve as useful input for any tracing algorithm.

This paper is a considerably extended version of our recent conference report (Radojević et al. 2014). We have modified the filtering algorithms and fuzzy-logic rules so as to be able to detect both junction and termination points. In addition we here present the full details of our method and an extensive evaluation based on both manually annotated real neuron images and computer generated neuron images. To obtain the latter we here propose a new computational approach based on publicly available expert manual tracings. We start with a brief overview of related work on critical-point detection (“Related Work”) and then present the underlying concepts (“Proposed Method”), implementational details (“Implementational Details”), and experimental evaluation (“Experimental Results”) of our method, followed by a summary of the conclusions that can be derived from the results (“Conclusions”).

## Related Work

Detecting topologically critical points in images has been a long-standing problem in many areas of computer vision. Although an in-depth review of the problem and proposed solutions is outside the scope of this paper, we provide a brief discussion in order to put our work into context.

Examples of previous work include the design of filters to find image locations where either three or more edges join (“junctions of edges”) (Sinzinger 2008; Hansen and Neumann 2004; Laganiere and Elias 2004) or three or more lines join (“junctions of lines”) (Yu et al. 1998; Deschênes and Ziou 2000). In biomedical applications, the predominant type of junction is the bifurcation, with occasional trifurcations, as seen in blood vessel trees, bronchial trees, gland ductal trees, and also in dendritic trees (Koene et al. 2009; Iber and Menshykau 2013). Hence, research in this area has focused on finding image locations where three (or more) elongated structures join (Tsai et al. 2004; Agam et al. 2005; Bevilacqua et al. 2005; Bhuiyan et al. 2007; Zhou et al. 2007; Aibinu et al. 2010; Calvo et al. 2011; Obara et al. 2012b; Su et al. 2012; Azzopardi and Petkov 2013).

A common approach to find bifurcation points is to infer them from an initial processing step that aims to segment the elongated structures. However, the way these structures are segmented may influence the subsequent critical-point inference. Popular image segmentation methods use intensity thresholding and/or morphological processing, in particular skeletonization (Hoover et al. 2000; Dima et al. 2002; He et al. 2003; Weaver et al. 2004; Pool et al. 2008; Bevilacqua et al. 2009; Leandro et al. 2009; Aibinu et al. 2010), but these typically produce very fragmented results. Popular methods to enhance elongated image structures prior to segmentation include Hessian based analysis (Frangi et al. 1998; Xiong et al. 2006; Zhang et al. 2007; Al-Kofahi et al. 2008; Yuan et al. 2009; Türetken et al. 2011; Myatt et al. 2012; Basu et al. 2013; Santamaría-Pang et al. 2015), Laplacean-of-Gaussian filters (Chothani et al. 2011), Gabor filters (Bhuiyan et al. 2007; Azzopardi and Petkov 2013), phase congruency analysis (Obara et al. 2012a), and curvelet based image filtering approaches (Narayanaswamy et al. 2011). However, being tailored to elongated structures, such filters often yield a less optimal response precisely at the bifurcation points, where the local image structure is more complex than a single ridge.

Several concepts have been proposed to explicitly detect bifurcation points in the images without relying on an initial segmentation of the axonal and dendritic trees. Examples include the usage of circular statistics of phase information (Obara et al. 2012b), steerable wavelet based local symmetry detection (Püspöki et al. 2013), or combining eigen analysis of the Hessian and correlation matrix (Su et al. 2012). The problem with existing methods is that they often focus on only one particular type of critical point (for example bifurcations but not terminations), or they use rather rigid geometrical models (for example assuming symmetry), while in practice there are many degrees of freedom (Michaelis and Sommer 1994). Image filtering methods for bifurcation detection have also been combined with supervised machine-learning based approaches such as support vector machines (Türetken et al. 2011), artificial neural networks (Bevilacqua et al. 2009), or with multiple classifiers using AdaBoost (Zhou et al. 2007), but these lack flexibility in that they require a training stage for each application.

Robust neuron tracing requires knowledge of not only the bifurcation points but also the termination points. Since each type of critical point may vary considerably in terms of geometry (orientation and diameter of the branches) and image intensity (often related to the branch diameter), designing or training a dedicated filter for each possible case is impractical, and a more integrated approach is highly desirable for both detection and characterization of the different types of critical points. To the best of our knowledge, no generic methods currently exist for critical-point detection in neuron images. The method proposed in this paper aims to fill this gap and to complement exploratory neuron reconstruction algorithms that initialize on a set of seed points.

## Proposed Method

Our proposed method for detection and characterization of critical points consists of three steps: feature extraction (“Feature Extraction”), fuzzy-logic based mapping (“Fuzzy-Logic Based Mapping”), and, finally, critical-point determination (“Critical-Point Determination”). Here we describe each step in detail.

### Feature Extraction

The aim of the feature extraction step is to compute a set of quantitative features of the local image structure at each pixel position that helps to discriminate between different types of critical points. Since the tree-like neuronal image structures typically cover only a small portion of the image, we avoid unnecessary computations by first performing a foreground selection step (“Foreground Selection”), which discards image locations that are very unlikely to contain neuronal structures and keeps only those regions that are worthy of further examination. Next, the angular profile (“Angular Profile Analysis”) of each foreground pixel is constructed, from which the quantitative features are computed.

**Fig. 2:**
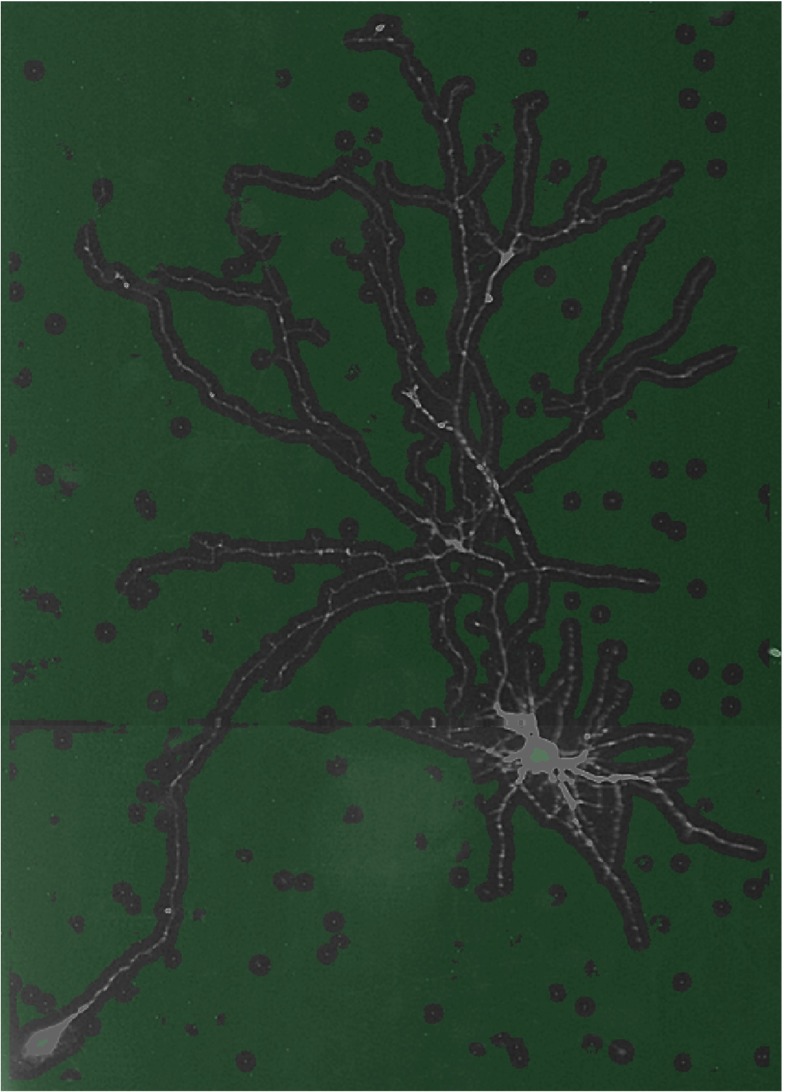
Example of foreground selection. The original image of 560×780 pixels is divided into background (*green transparent mask*) and foreground (*gray-scale regions without mask*) using *r*_*d*_ = 8 pixels and the 75^th^ variation percentile as threshold. In this example, 25% of the total number of pixels is selected for further processing

#### Foreground Selection

To determine whether a pixel location (*x*, *y*) in a given image *I* should be considered as foreground or background, we analyze the local intensity variation *ρ*(*x*, *y*) within a circular neighborhood of radius *r*_*d*_ centered at that location. To avoid making strong assumptions about the local intensity distribution we chose to use the difference between the 95^th^ and the 5^th^ percentile of the intensities within the neighborhood as the measure of variation:

1 $$\begin{array}{@{}rcl@{}} \rho(x,y) = \mathcal{P}_{95}(\mathcal{I}_{\!\!xy}) - \mathcal{P}_{5}(\mathcal{I}_{\!\!xy}) \end{array} $$

2 $$\begin{array}{@{}rcl@{}} \mathcal{I}_{\!\!xy} = \left\{\, I(m,n)\ |\ (m-x)^{2}+(n-y)^{2} \leq {r_{d}^{2}}\, \right\} \end{array} $$

3 $$\begin{array}{@{}rcl@{}} x,m \in [0,W-1]\ \text{and}\ y,n \in [0,H-1] \end{array} $$

where *W* and *H* denote, respectively, the width and the height of *I* in pixels. The value of *ρ* is relatively low within more or less homogeneous regions (background but also the soma) but relatively high in regions containing neuronal branches. Consequently, the histogram of *ρ* computed over the entire image contains two clusters (representing foreground and background pixels), which can be separated using simple percentile thresholding (Doyle 1962). The percentile should be chosen such that background pixels (true negatives) are removed as much as possible while at the same time the foreground pixels (true positives) are retained as much as possible (in practice this implies allowing for false positives). We found that in our applications a percentile of around 75 is a safe threshold (Fig. 2). Small gaps in the foreground region are closed by morphological dilation. The resulting set of foreground pixel locations is denoted by *F*. In our applications the parameter *r*_*d*_ is typically set to the diameter of the axonal and dendritic structures observed in the image.

#### Angular Profile Analysis

**Fig. 3:**
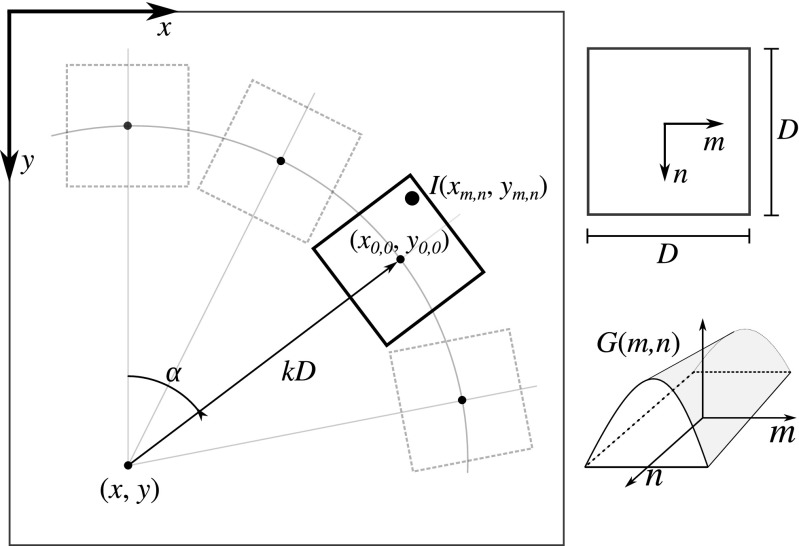
Geometry involved in the computation of the angular profile. In effect, the value of *p*(*x*, *y*, *α*, *k*, *D*) is the correlation of the image *I*(*x*, *y*) with the kernel *G*(*m*, *n*) of size *D*×*D* pixels, after rotating the kernel patch over angle *α* and shifting it over *k* *D* with respect to (*x*, *y*)

For each selected foreground location, a local angular profile is computed and analyzed. The key task here is to assess the presence and properties of any curvilinear image structures passing through the given location. To this end we correlate the image with a set of oriented kernels distributed evenly over a range of angles around that location (Radojević et al. 2014). The basic kernel used for this purpose is of size *D*×*D* pixels and has a constant profile in one direction and a Gaussian profile in the orthogonal direction (Fig. 3):

4 $$ G(x,y)=\mathrm{e}^{-x^{2}/2\sigma_{\!\!D}^{2}}/S $$

where *S* is a normalization factor such that the sum of *G*(*x*, *y*) over all kernel pixels is unity. We chose the Gaussian both because we observed that the cross-sectional profile of axons and dendrites in our applications is approximately Gaussian-like and because the Gaussian is a theoretically well-justified filter for regularization purposes. The parameters *D* and *σ*_*D*_ determine the size and shape of the kernel profile and should correspond to the expected branch diameter.

Using the kernel we compute the local angular profile at any pixel location (*x*, *y*) in the given image *I* as:

5 $$ p(x,y,\alpha,k,D)=\underset{m}{\sum}\underset{n}{\sum} I(x_{m,n},y_{m,n})\,G(m,n) $$

where the transformed image coordinates are obtained as:

6 $$ \left[\begin{array}{lllllll} x_{m,n} \\ y_{m,n} \end{array}\right] = \left[\begin{array}{lllllll} x \\ y \end{array}\right] + kD\left[\begin{array}{lllllll} \sin\alpha \\ -\!\cos\alpha \end{array}\right] + \left[\begin{array}{lllllll} \cos\alpha & -\!\sin\alpha \\ \sin\alpha & \cos\alpha \end{array}\right] \left[\begin{array}{lllllll} m \\ n \end{array}\right] $$

and the summation is performed over all (*m*, *n*) for which the kernel is defined. That is, *p*(*x*, *y*, *α*, *k*, *D*) is the correlation of the image with the kernel patch rotated over angle *α* and shifted over a distance *kD* with respect to (*x*, *y*) in the direction corresponding to that angle (Fig. 3). In practice, *p* is calculated for a discrete set of angles, *α*_*i*_ = *i*/(2*πN*_*α*_), *i* = 0,…, *N*_*α*_−1, where *N*_*α*_ is automatically set such that the circle with radius *kD* is sampled with pixel resolution. The parameter *k* is typically set slightly larger than 0.5 so as to scan the neighborhood around the considered pixel (*x*, *y*). To obtain the image intensity at non-integer transformed locations (*x*_*m*, *n*_, *y*_*m*, *n*_), linear interpolation is used.

In contrast with previous works, which used differential kernels for directional filtering and profiling (Yu et al. 1998; Can et al. 1999; Zhang et al. 2007), we employ the matched kernel (4), which avoids noise amplification. Although applying a set of rotated kernels is computationally more demanding than Hessian or steerable filtering based methods, it provides more geometrical flexibility in matching the kernels with the structures of interest while retaining excellent directional sensitivity. In our framework, the computational burden is drastically reduced by the foreground selection step, and further reduction is possible since the filtering process is highly parallelizable.

**Fig. 4:**
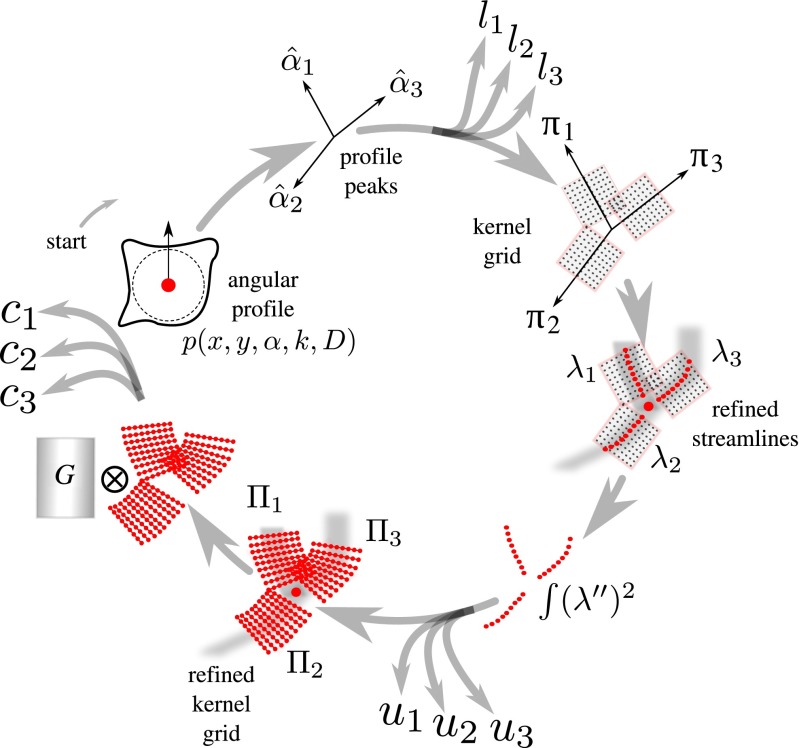
Flowchart of the feature extraction scheme. The example showcases a bifurcation but the same scheme is used also for terminations. The scheme, which starts with the angular profile *p*(*x*, *y*, *α*, *k*, *D*) and is executed clockwise, is applied to each pixel in the selected foreground regions and results in the set of features *l*_*i*_, *u*_*i*_, and *c*_*i*_, where *i* indexes the streamlines. See main text for details

After computing the angular profile we further process it in order to extract several features (Fig. 4) relevant for critical-point detection and characterization:

##### **Peaks**

At each foreground pixel location we first determine how many and in which direction line-like image structures pass through it. This is done by finding the local maxima (“peaks”) in the angular profile at that location. Since the oriented kernels act as low-pass filters, the profile is sufficiently smooth to extract the peaks reliably using the iterative line searching algorithm (Flannery et al. 1992). The found peaks correspond to angles $\hat {\alpha }_{i}, i=1,\dots ,N_{\hat {\alpha }}$, in which directions the image intensities are the highest. Here $N_{\hat {\alpha }}\leq 4$ to accommodate terminations, normal body points, and junctions (bifurcations and crossovers).

##### **Likelihood**

For each $\hat {\alpha }_{i}$ we calculate a likelihood *l*_*i*_∈[0,1] from the angular profile according to:

7 $$ l_{i} = \frac{p(x,y,\hat{\alpha}_{i},k,D)-p_{\min}}{p_{\max}-p_{\min}} $$

where *p*_min_ and *p*_max_ denote, respectively, the minimum and maximum of *p*(*x*, *y*, *α*, *k*, *D*) over *α*.

##### **Energy**

Next we consider the local grid ${\uppi }_{i}(x,y,\hat {\alpha }_{i},k,D)$ for each $\hat {\alpha }_{i}$ (Fig. 4), consisting of the transformed coordinates (*x*_*m*, *n*_, *y*_*m*, *n*_) corresponding to $\alpha =\hat {\alpha }_{i}$ (6), and we extract a refined centerline point set *λ*_*i*_ (or “streamline”) on this grid by finding for each *n* the local maximum over *m*:

8 $$\begin{array}{@{}rcl@{}} \lambda_{i} = \left\{(x_{\hat{m}_{n},n},y_{\hat{m}_{n},n})\right\}_{n\,\in\,\left[-D/2,D/2\right]} \end{array} $$

9 $$\begin{array}{@{}rcl@{}}[0.5ex] \hat{m}_{n} = \underset{m\,\in\,\left[-D/2,D/2\right]}{\arg\max} I(x_{m,n},y_{m,n}) \end{array} $$

We quantify how much the streamline deviates from a straight line by estimating its bending energy *u*_*i*_≥0 as:

10 $$ u_{i} = \frac{1}{\Updelta m}\underset{n}{\sum} \left( \hat{m}_{n-1} - 2\hat{m}_{n} + \hat{m}_{n+1} \right)^{2} $$

where Δ*m* is the pixel spacing in the direction of *m* and the summation extends over all *n* for which the summand can be evaluated. This calculation is a discrete approximation of the integral squared second-order derivative of the centerline function if it were continuously defined.

##### **Correlation**

Given a streamline *λ*_*i*_ we estimate the orthogonal direction at each point in the set by averaging the orthogonal directions of the two neighboring streamline segments corresponding to that point (that is, from the point to the next point, and from the point to the previous point). Using these direction estimates we sample a refined local grid $\Uppi _{i}(x,y,\hat {\alpha }_{i},k,D)$, consisting of image coordinates $(\tilde {x}_{m,n},\tilde {y}_{m,n})$ relative to the streamline (Fig. 4), and compute a normalized cross-correlation (Lewis 1995) score *c*_*i*_∈[−1,1] as:

11 $$ c_{i} = \frac{{\sum}_{m}{\sum}_{n} \left[I(\tilde{x}_{m,n},\tilde{y}_{m,n})-\bar{I}\right] \left[G(m,n)-\bar{G}\right]}{\sqrt{{\sum}_{m}{\sum}_{n}\left[I(\tilde{x}_{m,n},\tilde{y}_{m,n})-\bar{I}\right]^{2}{\sum}_{m}{\sum}_{n}\left[G(m,n)-\bar{G}\right]^{2}}} $$

where, similar to the angular profile calculation (5), the summations extend over all (*m*, *n*) for which the kernel is defined, and $\bar {I}$ and $\bar {G}$ denote the mean of the image intensities and of the kernel values, respectively. Effectively *c*_*i*_ quantifies the degree to which the template *G* matches a straightened version of the streamline. To cover a range of possible scales (radii of the underlying image structures), we take the largest score of a set of templates with standard deviations of the Gaussian profile model (Su et al. 2012) covering $\left \lbrace 1,\dots ,\left \lfloor {\frac {D}{2}}\right \rfloor \right \rbrace $ set of values measured in pixels.

### Fuzzy-Logic Based Mapping

The feature values extracted at each foreground image location subsequently need to be processed in order to assess the presence of a critical point and its type. Recognizing that in practice everything is “a matter of degree” (Zadeh 1975), and allowing for nonlinear input-output mappings, we chose to use fuzzy logic for this purpose. Fuzzy logic has been successfully used in many areas of engineering (Mendel 1995) but to the best of our knowlege has not been explored for neuron critical-point analysis. We briefly describe the basics of fuzzy logic (“Basics of Fuzzy Logic”) and then present our specific fuzzy-logic system for calculating critical-point maps of neuron images (“Termination and Junction Mapping”).

#### Basics of Fuzzy Logic

**Fig. 5:**
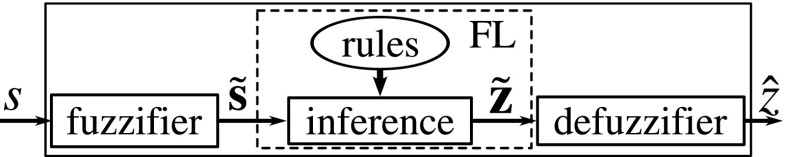
Scheme of a single input/output fuzzy-logic (FL) system. A scalar input value *s* is converted to a vector $\tilde {\mathbf {s}}$ containing the memberships of *s* for each of the input fuzzy sets, resulting in a vector $\tilde {\mathbf {z}}$ containing the memberships of *z* for each of the output fuzzy sets

In a fuzzy-logic system (Fig. 5), numerical inputs are first expressed in linguistic terms (the fuzzification step), and are then processed based on predefined rules to produce linguistic outputs (the inference step), which are finally turned back into numerical values (the defuzzification step).

##### **Fuzzification**

Given an input scalar value $s\in \mathbb {R}$, the fuzzification step results in a vector $\tilde {\mathbf {s}}$ whose elements express the degree of membership of *s* to input fuzzy sets, each corresponding to a linguistic term describing *s*. A fuzzy set is defined by a membership function $\mu :\mathbb {R}\rightarrow [0,1]$ quantifying the degree to which *s* can be described by the corresponding linguistic term. Commonly used membership functions are trapezoidal, Gaussian, triangular, and piecewise linear (Mendel 1995). As an example, we may have linguistic terms LOW and HIGH, representing the subjective notions “low” and “high”, respectively. The degrees to which “*s* is low” (which in this paper we will write as *s* = LOW) and “*s* is high” (*s* = HIGH) are given by membership values *μ*_LOW_(*s*) and *μ*_HIGH_(*s*), respectively. The output of the fuzzification step thus becomes $\tilde {\mathbf {s}}=[\mu _{\text {LOW}}(s),\mu _{\text {HIGH}}(s)]^{T}$.

##### **Inference**

The input fuzzy set memberships are processed by the inference engine to produce a fuzzy output based on rules expressing expert knowledge. The rules can be either explicitly defined or implicitly learned by some training process, and may express nonlinear input-output relationships and involve multiple inputs. In engineering applications, the rules are commonly given as IF-THEN statements about the input and output linguistic terms. For example, the output terms could be OFF, NONE, and ON, indicating whether a certain property of interest is “off”, “none” (expressing ambiguity), or “on”. A rule could then be:

12 $$ \begin{array}{lllllll} R_{i}\!:\ \ & \text{IF}\ (s_{1}=\text{HIGH})\ \wedge\ (s_{2}=\text{LOW}) \\ & \text{THEN}\ (z=\text{OFF}) \end{array} $$

where $z\in \mathbb {R}$ is the variable over the output range. This is not a binary logical statement, where the input and output conditions can be only true or false, but a fuzzy logical statement, where the conditions are expressed in terms of memberships, in this case *μ*_HIGH_(*s*_1_), *μ*_LOW_(*s*_2_), and *μ*_OFF_(*z*). Input conditions are often combined using the operators ∧ (denoting fuzzy intersection) or ∨ (denoting fuzzy union), which are commonly defined as, respectively, the minimum and maximum of the arguments (Mendel 1995). In our example, the IF-part of *R*_*i*_ (12) would result in the following intermediate value (degree of verity):

13 $$ \upsilon_{i} = \min\left\{\mu_{\text{HIGH}}(s_{1}),\mu_{\text{LOW}}(s_{2})\right\} $$

This value is then used to constrain the fuzzy set corresponding to the output linguistic term addressed by *R*_*i*_, in this case OFF, resulting in the output fuzzy set:

14 $$ {\Upsilon}_{\!i}(z) = \min\left\{\mu_{\text{OFF}}(z),\upsilon_{i}\right\} $$

In practice there may be many rules *R*_*i*_, *i* = 1,…, *N*_*R*_, which are aggregated by the inference engine to produce a single output fuzzy set Υ. The common way to do this (Mendel 1995) is by means of a weighted fuzzy union:

15 $$ {\Upsilon}(z) = \max\left\{w_{1}{\Upsilon}_{\!1}(z),\dots,w_{N_{R}}{\Upsilon}_{\!N_{R}}(z)\right\} $$

Although it is possible to assign a different weight to each rule by setting *w*_*i*_∈[0,1], in our applications this is not critical, and therefore we simply use *w*_*i*_ = 1 for all *i*.

##### **Defuzzification**

In the final step of the fuzzy-logic system, the fuzzy output Υ is converted back to a scalar output value. Although there are many ways to do this, a common choice is to calculate the centroid (Mendel 1995):

16 $$ \hat{z} = \frac{\int z{\Upsilon}(z)dz}{\int{\Upsilon}(z)dz} $$

With this value we can finally calculate the vector of output fuzzy set memberships: $\tilde {\mathbf {z}}=[\mu _{\text {OFF}}(\hat {z}), \mu _{\text {NONE}}(\hat {z}), \mu _{\text {ON}}(\hat {z})]^{T}$.

#### Termination and Junction Mapping

**Fig. 6:**
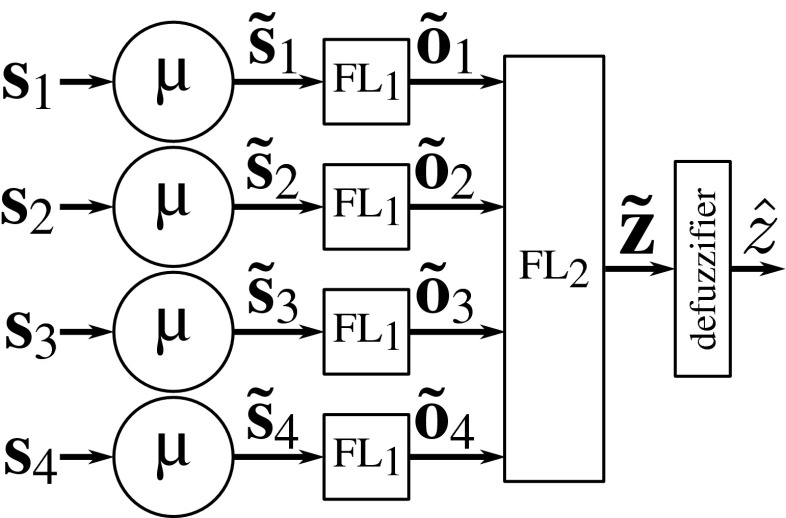
Architecture of the proposed fuzzy-logic system for critical-point detection. A cascade of two fuzzy-logic modules (FL_1_ and FL_2_) is used, where the first determines the degree to which streamlines (up to four) are present at the image location under consideration, and based on this information the second determines the degree to which that location corresponds to the possible types of critical points

**Fig. 7:**
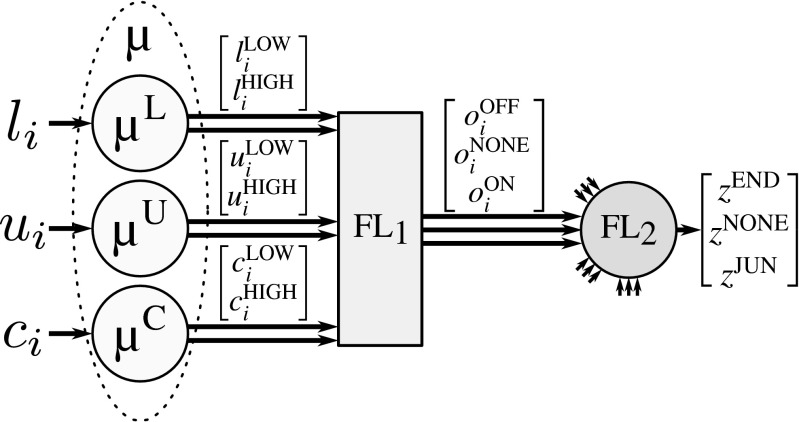
Architecture of the proposed fuzzy-logic system for processing the information of one streamline. Input feature values are fuzzified into linguistic terms LOW and HIGH, which are translated by the first fuzzy-logic module (FL_1_) into intermediate linguistic terms OFF, NONE, ON, which are finally translated by the second fuzzy-logic module (FL_2_) into linguistic terms END, NONE, JUN

To determine the presence and type of critical point at any foreground image location, we use a cascade of two fuzzy-logic systems, representing two decision levels (Fig. 6). The first level takes as input vectors **s**_*i*_ = [*l*_*i*_, *u*_*i*_, *c*_*i*_], *i* = 1,…,4, which contain the features for each of the streamlines extracted in the angular profile analysis step at the image location under consideration (“Angular Profile Analysis”). For each streamline (Fig. 7), the features are fuzzified (*μ*) and processed by the first fuzzy-logic module (FL_1_), which determines the degree to which the streamline indeed represents a line-like image structure (ON), or not (OFF), or whether the image structure is ambiguous (NONE). In cases where less than four streamlines were found by the angular profile analysis step, the feature vectors of the nonexisting streamlines are set to **0**. The fuzzy output for all four streamlines together forms the input for the second decision level, where another fuzzy-logic module (FL_2_) determines the degree to which the image location corresponds to a junction (JUN), or a termination (END), or neither of these (NONE).

**Fig. 8:**
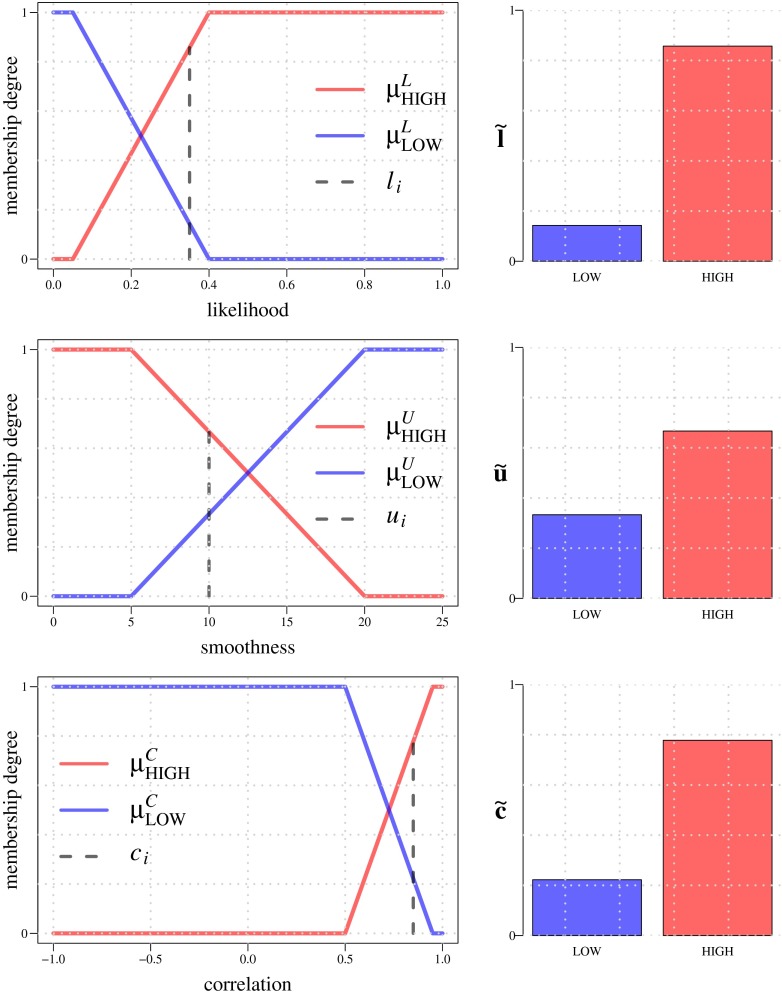
Input membership functions used in the fuzzification step for FL_1_. Example LOW and HIGH membership values are shown (*right column*) for input values (*dashed vertical lines in the plots on the left*) *l*_*i*_ = 0.35 (*top row*), *u*_*i*_ = 10 (*middle row*), and *c*_*i*_ = 0.85 (*bottom row*). The inflection points of the membership functions are, respectively, *L*_LOW_ = 0.05 and *L*_HIGH_ = 0.4 for *μ* ^*L*^, *U*_HIGH_ = 5 and *U*_LOW_ = 20 for *μ* ^*U*^, and *C*_LOW_ = 0.5 and *C*_HIGH_ = 0.95 for *μ* ^*C*^. Notice that features *u*_*i*_ (the centerline bending energies of the streamlines) are reinterpreted here to express the degree of smoothness (hence the inverted membership functions as compared to the other two)

The input streamline features, *l*_*i*_, *u*_*i*_, *c*_*i*_, are expressed in linguistic terms LOW and HIGH using membership functions *μ*_LOW_ and *μ*_HIGH_ defined for each type of feature. In our application we use trapezoidal membership functions, each having two inflection points, such that *μ*_LOW_ and *μ*_HIGH_ are each other’s complement (Fig. 8). For example, the degrees to which *l*_*i*_ = LOW and *l*_*i*_ = HIGH, are given by $l_{i}^{\text {LOW}}=\mu ^{L}_{\text {LOW}}(l_{i})$ and $l_{i}^{\text {HIGH}}=\mu ^{L}_{\text {HIGH}}(l_{i})=1-l_{i}^{\text {LOW}}$, respectively, and because of this complementarity we often simply write *μ*^*L*^ to refer to both membership functions (Fig. 7). Similarly, the membership degrees of *u*_*i*_ and *c*_*i*_ are given by *μ*^*U*^ and *μ*^*C*^, respectively. Summarizing, we use the following notations and definitions for the fuzzification step:

17 $$ \begin{array}{lcccl} \mu^{L}\!:\ & l_{i} & \rightarrow & \tilde{\mathbf{l}}_{i} & = \left[l_{i}^{\text{LOW}}\!,\, l_{i}^{\text{HIGH}}\right]^{T} \\[1ex] \mu^{U}\!:\ & u_{i} & \rightarrow & \tilde{\mathbf{u}}_{i} & = \left[u_{i}^{\text{LOW}}\!,\, u_{i}^{\text{HIGH}}\right]^{T} \\[1ex] \mu^{C}\!:\ & c_{i} & \rightarrow & \tilde{\mathbf{c}}_{i} & = \left[c_{i}^{\text{LOW}}\!,\, c_{i}^{\text{HIGH}}\right]^{T} \end{array} $$

and the lower and higher inflection points of *μ*^*L*^ are denoted by *L*_LOW_ and *L*_HIGH_, and similarly *U*_LOW_ and *U*_HIGH_ for *μ*^*U*^, and *C*_LOW_ and *C*_HIGH_ for *μ*^*C*^ (Fig. 8).

Taken together, the input to FL_1_ is the matrix of memberships $\tilde {\mathbf {s}}_{i}=[\tilde {\mathbf {l}}_{i},\tilde {\mathbf {u}}_{i},\tilde {\mathbf {c}}_{i}]$, and the output is the vector $\tilde {\mathbf {o}}_{i}$ of memberships to the linguistic terms OFF, NONE, ON:

18 $$ \text{FL}_{1}\!:\ \tilde{\mathbf{s}}_{i} \rightarrow \tilde{\mathbf{o}}_{i} = \left[o_{i}^{\text{OFF}}\!,\, o_{i}^{\text{NONE}}\!,\, o_{i}^{\text{ON}}\right]^{T} $$

**Fig. 9:**
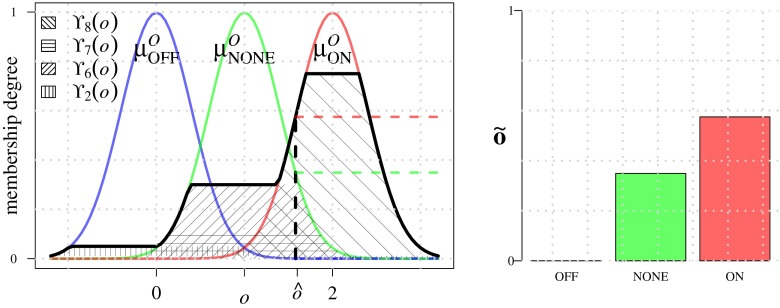
Output membership functions used in module FL_1_. Example output fuzzy sets Υ_*i*_ corresponding to rules *R*_*i*_ from Table 1 are shown as the textured areas. Value $\hat {o}$ (*left panel*) represents the centroid of the aggregated output fuzzy sets. The resulting output membership values (*right panel*) serve as input for module FL_2_

To calculate these memberships we introduce scalar variable *o*, where *o* = 0 corresponds to OFF, *o* = 1 to NONE, and *o* = 2 to ON, and we define Gaussian membership functions $\mu _{\text {OFF}}^{O}$, $\mu _{\text {NONE}}^{O}$ and $\mu _{\text {ON}}^{O}$, centered around 0, 1, and 2, respectively (Fig. 9), and with fixed standard deviation 0.4 so that they sum to about 1 in the interval [0,2]. The rules used by FL_1_ to associate the input terms LOW and HIGH to the output terms OFF, NONE, and ON, are given in Table 1. They are based on the heuristic assumption that a line-like image structure exists (ON) if the evidence represented by all three features support it (HIGH). By contrast, if the likelihood is LOW and at least one other feature is also LOW, this indicates that no such structure exists (OFF). In all remaining cases, some structure may exist, but it is not line-like (NONE). As an example, rule *R*_8_ (Table 1) is given by:

19 $$\begin{array}{@{}rcl@{}} R_{8}\!:\ \ &\text{IF}&\ (l=\text{HIGH})\ \wedge\ (u=\text{HIGH})\ \wedge\ (c=\text{HIGH})\\[-2pt] &&\text{THEN}\ (o = \text{ON}) \end{array} $$

which results in the verity value:

20 $$ \upsilon_{8} = \min\left\{\mu_{\text{HIGH}}^{L}(l), \mu_{\text{HIGH}}^{U}(u), \mu_{\text{HIGH}}^{C}(c)\right\} $$

and the output fuzzy set:

21 $$ {\Upsilon}_{8}(o) = \min\left\{\mu_{\text{ON}}^{O}(o),\upsilon_{8}\right\} $$

All the rules are resolved and combined as:

22 $$ {\Upsilon}(o) = \max\left\{{\Upsilon}_{1}(o),\dots,{\Upsilon}_{8}(o)\right\} $$

and centroid defuzzification then results in a scalar output value $\hat {o}$. This procedure is repeated for each streamline, yielding $\hat {o}_{i}, i=1,\dots ,4$, from which the output of each FL_1_ (18) is calculated using the membership functions:

23 $$ \tilde{\mathbf{o}}_{i} = \left[\mu_{\text{OFF}}^{O}(\hat{o}_{i}),\, \mu_{\text{NONE}}^{O}(\hat{o}_{i}),\, \mu_{\text{ON}}^{O}(\hat{o}_{i})\right]^{T} $$

**Table 1:** The set of rules employed by FL_1_

*R*_*i*_	*l*	*u*	*c*	*o*
1	LOW	LOW	LOW	OFF
2	LOW	LOW	HIGH	OFF
3	LOW	HIGH	LOW	OFF
4	LOW	HIGH	HIGH	NONE
5	HIGH	LOW	LOW	NONE
6	HIGH	LOW	HIGH	NONE
7	HIGH	HIGH	LOW	NONE
8	HIGH	HIGH	HIGH	ON

Moving on to the next level, the input to FL_2_ is the matrix of memberships $\tilde {\mathbf {o}}=\left [\tilde {\mathbf {o}}_{1},\tilde {\mathbf {o}}_{2},\tilde {\mathbf {o}}_{3},\tilde {\mathbf {o}}_{4}\right ]$, and the output is the vector $\tilde {\mathbf {z}}$ of memberships to the linguistic terms END (termination), NONE (no critical point), JUN (junction):

24 $$ \text{FL}_{2}\!:\ \tilde{\mathbf{o}} \rightarrow \tilde{\mathbf{z}} = \left[z^{\text{END}}\!,\, z^{\text{NONE}}\!,\, z^{\text{JUN}}\right]^{T} $$

**Fig. 10:**
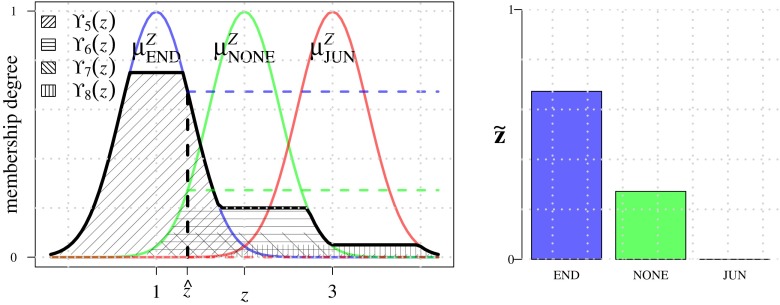
Output membership functions used in module FL_2_. Example output fuzzy sets Υ_*i*_ corresponding to rules *R*_*i*_ from Table 2 are shown as the textured areas. Value $\hat {z}$ (*left panel*) represents the centroid of the aggregated output fuzzy sets. The resulting output membership values (*right panel*) indicate the degree to which there may be a termination (END), junction (JUN), or neither of these (NONE) at the image pixel location under consideration

To calculate these memberships we introduce scalar variable *z*, where *z* = 1 corresponds to END, *z* = 2 to NONE, and *z* = 3 to JUN, and we define corresponding Gaussian membership functions $\mu _{\text {END}}^{Z}$, $\mu _{\text {NONE}}^{Z}$, and $\mu _{\text {JUN}}^{Z}$, centered around 1, 2, and 3, respectively, and with fixed standard deviation 0.4 as before (Fig. 10). The rules used by FL_2_ to associate the input terms OFF, NONE, ON to the output terms END, NONE, JUN are given in Table 2. They are based on the heuristic assumption that there is a termination (END) if a single streamline is confirmed to correspond to a line-like image structure (ON) and the other three are confirmed to not correspond to such a structure (OFF). Conversely, if at least three are ON, there must be a junction at that location. Finally, if two are ON and two are OFF, or if at least two streamlines are ambiguous (NONE), we assume there is no critical point. Similar to FL_1_, all the rules of FL_2_ are evaluated and their results combined as:

25 $$ {\Upsilon}(z) = \max\left\{{\Upsilon}_{1}(z),\dots,{\Upsilon}_{22}(z)\right\} $$

which, after centroid defuzzification, results in a scalar output value $\hat {z}$, from which the output of FL_2_ (24) is calculated using the membership functions:

26 $$ \tilde{\mathbf{z}} = \left[\mu_{\text{END}}^{Z}(\hat{z}),\, \mu_{\text{NONE}}^{Z}(\hat{z}),\, \mu_{\text{JUN}}^{Z}(\hat{z})\right]^{T} $$

The proposed fuzzy-logic system is applied to each foreground pixel location (*x*, *y*)∈*F* (“Foreground Selection”) so that all memberships introduced above may be indexed by (*x*, *y*). Based on this we calculate the following two maps:

27 $$ I_{\text{END}}(x,y)=\left\{ \begin{array}{l@{\qquad}l} z^{\text{END}}(x,y) & \text{if}\ (x,y)\in F \\ 0 & \text{otherwise} \end{array} \right. $$

28 $$ I_{\text{JUN}}(x,y)=\left\{ \begin{array}{l@{\qquad}l} z^{\text{JUN}}(x,y) & \text{if}\ (x,y)\in F \\ 0 & \text{otherwise} \end{array} \right. $$

which indicate the degree to which any pixel (*x*, *y*) belongs to a termination or a junction, respectively.

**Table 2:** The set of rules employed by FL_2_. Empty entries indicate “don’t care” (could be OFF, NONE, or ON)

*R*_*i*_	*o*_1_	*o*_2_	*o*_3_	*o*_4_	*z*
1	OFF	OFF	OFF	OFF	NONE
2	OFF	OFF	OFF	ON	END
3	OFF	OFF	ON	OFF	END
4	OFF	OFF	ON	ON	NONE
5	OFF	ON	OFF	OFF	END
6	OFF	ON	OFF	ON	NONE
7	OFF	ON	ON	OFF	NONE
8	OFF	ON	ON	ON	JUN
9	ON	OFF	OFF	OFF	END
10	ON	OFF	OFF	ON	NONE
11	ON	OFF	ON	OFF	NONE
12	ON	OFF	ON	ON	JUN
13	ON	ON	OFF	OFF	NONE
14	ON	ON	OFF	ON	JUN
15	ON	ON	ON	OFF	JUN
16	ON	ON	ON	ON	JUN
17	NONE	NONE			NONE
18	NONE		NONE		NONE
19	NONE			NONE	NONE
20		NONE	NONE		NONE
21		NONE		NONE	NONE
22			NONE	NONE	NONE

### Critical-Point Determination

The ultimate aim of our method is to provide a list of critical points in the neuron image, with each point fully characterized in terms of type, location, size, and main branch direction(s). Since each critical point of a neuronal tree typically covers multiple neighboring pixels in the image, giving rise to a high value at the corresponding pixels in the maps *I*_END_ and *I*_JUN_, the final task is to segment the maps and to aggregate the information within each segmented region.

Due to noise, labeling imperfections, and structural ambiguities in the original image, the values of neighboring pixels in the maps may vary considerably, and direct thresholding usually does not give satisfactory results. To improve the robustness we first regularize the real-valued scores in the maps by means of local-average filtering with a radius of 3-5 pixels. Next, max-entropy based automatic thresholding (Kapur et al. 1985) is applied to segment the maps, as in contrast with many other thresholding methods we found it to perform well in separating the large but relatively flat (low information) background regions from the much smaller but more fluctuating (high information) regions of interest. The resulting binary images are further processed using a standard connected components algorithm (Sonka et al. 2007) to identify the critical-point regions.

**Fig. 11:**
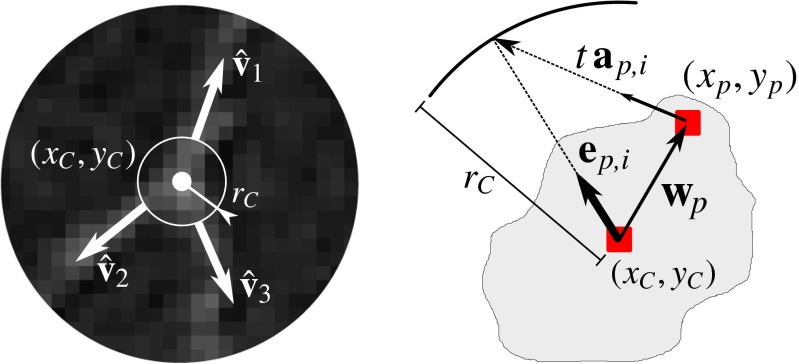
Critical-point determination. A critical point is characterized by its type, centroid location (*x*_*C*_, *y*_*C*_), radius *r*_*C*_, and its main branch directions $\hat {\mathbf {v}}_{i}$ (*left panel*, in this case a bifurcation), aggregated from the pixels (*x*_*p*_, *y*_*p*_) in the corresponding critical region (*right panel*)

Each critical region consists of a set of connected pixels **x**_*p*_ = (*x*_*p*_, *y*_*p*_), *p* = 1,…, *N*_*p*_, where *N*_*p*_ denotes the number of pixels in the region. From these, the representative critical-point location **x**_*C*_ = (*x*_*C*_, *y*_*C*_) is calculated as:

29 $$ \mathbf{x}_{C}=\frac{1}{N_{p}}\sum\limits_{p=1}^{N_{p}}\mathbf{x}_{p} $$

while the critical-point size is represented by the radius of the minimum circle surrounding the region:

30 $$ r_{C} = \max_{p}\left\{||\mathbf{w}_{p}||\right\} $$

where **w**_*p*_ = **x**_*p*_−**x**_*C*_ (Fig. 11). To obtain regularized estimates of the main branch directions $\hat {\mathbf {v}}_{i}$ for the critical point, we aggregate the directions corresponding to the angular profile peaks $\hat {\alpha }_{i}$ (“Angular Profile Analysis”) of all the **x**_*p*_ in the region as follows. For each **x**_*p*_ we have $N_{\hat {\alpha }}\leq 4$ angular profile peak direction vectors $\mathbf {a}_{p,i}=[\cos \hat {\alpha }_{i}(\mathbf {x}_{p}),\sin \hat {\alpha }_{i}(\mathbf {x}_{p})]^{T}$. Each of these vectors defines a line **a**(*t*) = **x**_*p*_ + *t***a**_*p*, *i*_ parameterized by $t\in \mathbb {R}$. We establish the projection of this line onto the circle $||\mathbf {x}-\mathbf {x}_{C}||^{2}={r^{2}_{C}}$ by substituting **x** = **a**(*t*) and solving for *t*. From this we calculate the contributing unit vector (Fig. 11):

31 $$ \mathbf{e}_{p,i}=\frac{1}{r_{C}}(\mathbf{w}_{p}+t\,\mathbf{a}_{p,i}) $$

which points from **x**_*C*_ to the intersection point. This is done for all *p* = 1,…, *N*_*p*_ in the region and $i=1,\dots ,N_{\hat {\alpha }}$ for each *p*, resulting in the set of vectors {**e**_*p*, *i*_}. Next, a recursive mean-shift clustering algorithm (Cheng 1995) is applied to {**e**_*p*, *i*_}, which converges to a set $\{\hat {\mathbf {v}}_{i}\}$, where the cluster vectors $\hat {\mathbf {v}}_{i}$, *i* = 1,…, *L*, represent the branches. For a critical region in *I*_END_, we need only one main branch direction, which we simply take to be the direction $\hat {\mathbf {v}}_{1}$ to which the largest number of **e**_*p*, *i*_ were shifted. For a critical region in *I*_JUN_, we take as the main branch directions the $\hat {\mathbf {v}}_{i}$ (at least three) to which the largest number of **e**_*p*, *i*_ were shifted. These calculations are performed for all critical regions.

## Implementational Details

The method was implemented in the Java programming language as a plugin for the image processing and analysis tool ImageJ (Abràmoff et al. 2004; Schneider et al. 2012). Since the feature extraction step (“Feature Extraction”), in particular the matched filtering for angular profile analysis, is quite computationally demanding, we applied parallelization in multiple ways to reduce the running time to acceptable levels (on the order of minutes on a regular PC). Specifically, the directional filtering was split between CPU cores, each taking care of a subset of the directions (depending on the number of available cores). After this, the angular profile analysis and calculation of the features was also split, with each core processing a subset of the foreground image locations. This was sufficient for our experiments. Further improvement in running time (down to real-time if needed) could be achieved by mass parallelization using GPUs (graphical processing units) instead of CPUs.

Essential parameters that need to be set by the user are the scale parameters *k* and *D* (“Angular Profile Analysis”) and the inflection points *L*_LOW_, *L*_HIGH_, *U*_LOW_, *U*_HIGH_, *C*_LOW_, and *C*_HIGH_ of the input membership functions used by fuzzy-logic module FL_1_ (“??”). In our applications we set *D* to the expected neuron diameter in a given set of images while *k* = 0.7 was kept fixed. The *L* inflection points are always in the range [0,1] since the corresponding feature (likelihood) is normalized. Based on ample experience with many data sets we typically set *L*_LOW_ close to 0 and *L*_HIGH_ around 0.5 (Fig. 8). By contrast, the inflection points *U* correspond to a feature (centerline bending energy) that is not normalized and may vary widely from 0 to any positive value. To obtain sensible values for these we rely on the histogram of all calculated energy values in the image. Parameter *U*_LOW_ is set to the threshold computed by the well-known triangle algorithm, while typically *U*_HIGH_≫*U*_LOW_. We note that the membership functions defined by these parameters are inverted (Fig. 8) such that the energy becomes a measure of smoothness. Finally, the *C* inflection points correspond again to a feature (correlation) with a fixed output range [−1,1]. In our applications we usually set them to *C*_LOW_∈[0.1,0.5] and *C*_HIGH_ = 0.95 (Fig. 8).

All other aspects of our method that could be considered as user parameters either follow directly from these essential parameters or are fixed to the standard values mentioned in the text. For example, the radius *r*_*d*_ of the circular neighborhood in the foreground selection step (“Foreground Selection”) can be set equal to *D*, and the standard deviation *σ*_*D*_ of the Gaussian profile (“Angular Profile Analysis”) can be set to *D*/6 to get a representative shape. Also, the output membership functions of FL_1_ (input to FL_2_) as well as the output membership functions of FL_2_ are Gaussians with fixed levels and standard deviation (“Termination and Junction Mapping”), as they are not essentially influencing the performance of the algorithm.

## Experimental Results

To evaluate the performance of our method in correctly detecting and classifying neuronal critical points we performed experiments with simulated images (using the ground truth available from the simulation) as well as with real fluorescence microscopy images (using manual annotation as the gold standard). After describing the performance measures (“Performance Measures”), we present and discuss the results of the evaluation on simulated images, including synthetic triplets (“Evaluation on Simulated Triplet Images”) and neurons (“Evaluation on Simulated Neuron Images”), and on real neuron images (“Evaluation on Real Neuron Images”), as well as the results of a comparison of our method with two other methods (“Comparison With Other Methods”).

### Performance Measures

Performance was quantified by counting the correct and incorrect hits and the misses of the detection with respect to the reference data. More specifically, we counted the true-positive (TP), false-positive (FP), and the false-negative (FN) critical-point detections, and we used these to calculate the recall R=TP/(TP+FN) and precision P=TP/(TP+FP). Both R and P take on values in the range from 0 (meaning total failure) to 1 (meaning flawless detection). They are commonly combined in the F-measure (Powers 2011), defined as the harmonic mean of the two: F=2 R P/(R+P). The F-measure was computed separately for each type of critical points considered in this paper, yielding F_END_ for terminations and F_JUN_ for junctions. As a measure of overall performance we also computed the harmonic mean of the two F-measures: F_BOTH_ = 2 F_END_ F_JUN_/(F_END_+F_JUN_).

### Evaluation on Simulated Triplet Images

**Fig. 12:**
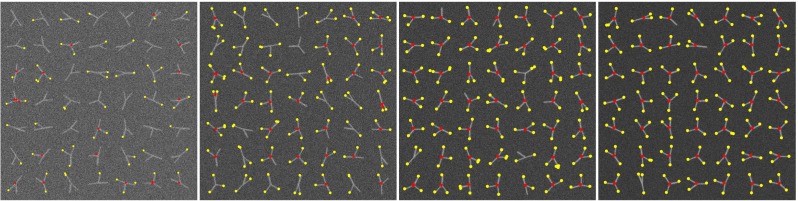
Examples of simulated triplet images and detection results. Each triplet consists of three branches with different diameters which join at one end to form a bifurcation point and with the other ends being termination points. Images were generated at SNR=2,3,4,5 (*left* to *right* panel). The detection results with our method are indicated as red circles (bifurcation points) and yellow circles (termination points), where the radius of each circle reflects the size of the critical region found by our method

**Fig. 13:**
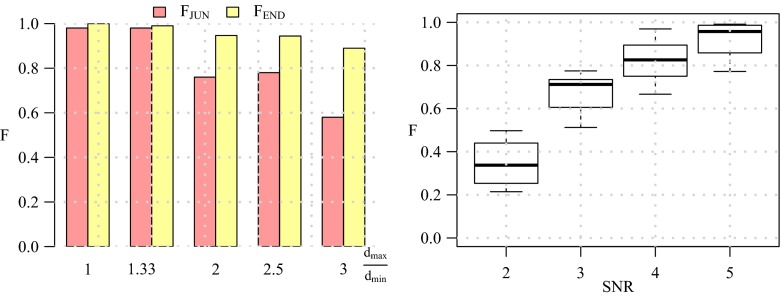
Performance of our method in detecting terminations and junctions in simulated images of triplets. The values of F_END_ and F_JUN_ are shown (*left panel*) for the various branch diameter ratios $d_{\max }/d_{\min }$ at SNR=4. The distribution of F_BOTH_ values is shown as a box plot (*right panel*) for the various SNR levels

Before considering full neuron images we first evaluated the performance of our method in detecting terminations and junctions in a very basic configuration as a function of image quality. To this end we used a triplet model, consisting of a single junction modeling a bifurcation, having three branches with arbitrary orientations (angular intervals) and diameters (Fig. 12). Orientations were randomly sampled from a uniform distribution in the range [0,2*π*] while prohibiting branch overlap. Since in principle the directional filtering step (“Angular Profile Analysis”) uses a fixed kernel size *D*, we wanted to investigate the robustness of the detection for varying ratios *d*_max_/*d*_min_ between the maximum and the minimum branch diameter in a triplet. For this experiment we considered ratios 1,0.33,2,2.5,3 by taking normalized diameter configurations (*d*_1_, *d*_2_, *d*_3_) = (0.33,0.33,0.33), (0.3,0.3, 0.4), (0.2, 0.4,0.4), (0.2,0.3,0.5), (0.2,0.2,0.6), where in each case the actual smallest diameter was set to 3 pixels (the resolution limit) and the other diameters were scaled accordingly. For each configuration we simulated images with 1,000 well-separated triplets for signal-to-noise ratio levels SNR=2, 3, 4, 5 (see cropped examples in Fig. 12). We chose these levels knowing that SNR=4 is a critical level in other detection problems (Smal et al. 2010; Chenouard et al. 2014). Poisson noise was used in simulating fluorescence microscopy imaging of the triplets. From the results of this experiment (Fig. 13) we conclude that our method is very robust for diameter ratios $d_{\max }/d_{\min }\leq 2\frac {1}{2}$ and an image quality of SNR≥4. We also conclude that our method is somewhat better in detecting terminations than detecting junctions. Example detection results for *d*_max_/*d*_min_≤2 for the considered SNR levels are shown in Fig. 12.

### Evaluation on Simulated Neuron Images

**Fig. 14:**
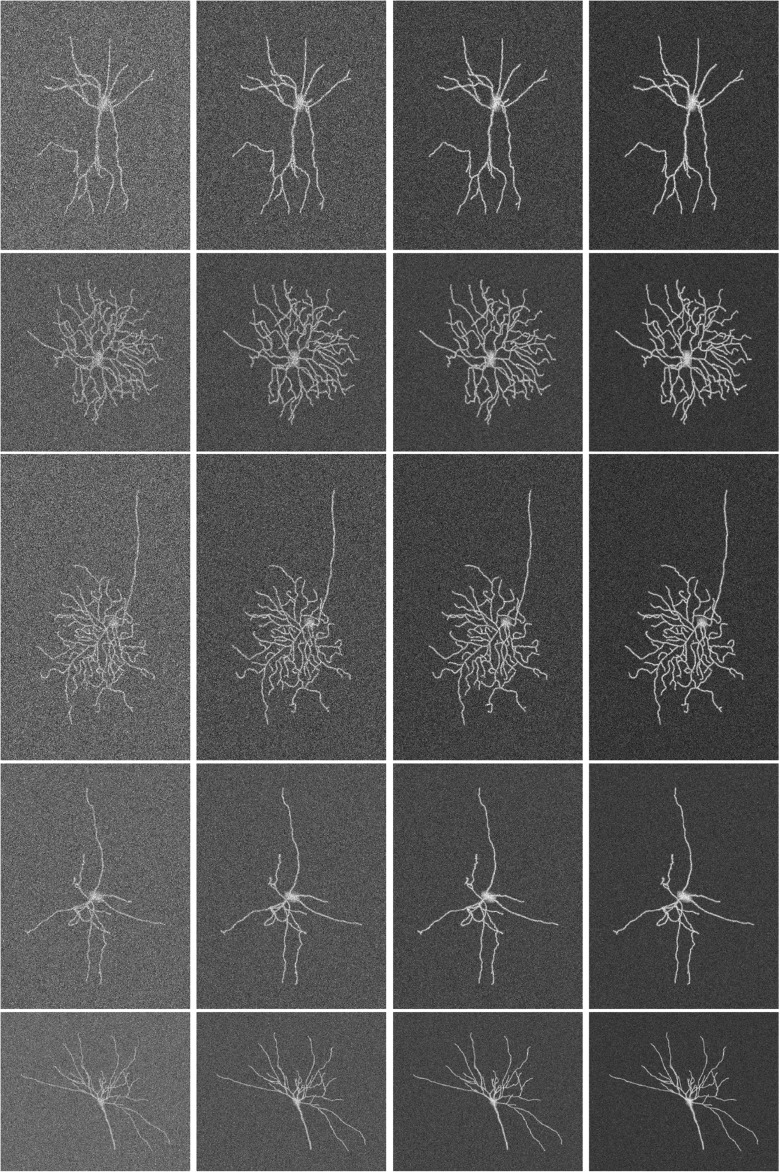
Examples of simulated neuron images based on expert reconstructions from the NeuroMorpho.Org database. The images show a wide range of morphologies (one type per row) and image qualities of SNR=2, 3, 4, 5 (from *left* to *right* per row)

To evaluate our method on more complex images, but for which we would still know the truth exactly, we simulated the imaging of complete neurons. Although there are various ways this could be done (Koene et al. 2009; Vasilkoski and Stepanyants 2009), we chose to use existing expert reconstructions from the NeuroMorpho.Org database (Ascoli et al. 2007). A total of 30 reconstructions from different neuron types were downloaded as SWC files (Cannon et al. 1998), in which the reconstructions are represented as a sequence of connected center-point locations in 3D with corresponding radii in micrometers. Fluorescence microscopy images were generated from these reconstructions in 2D by using a Gaussian point-spread function model and Poisson noise to emulate diffraction-limited optics and photon statistics. For each reconstruction we generated images of SNR=2, 3, 4, 5 (Fig. 14). This way we obtained simulated images of neurons for which the termination and junction point locations were known exactly from the SWC files.

**Fig. 15:**
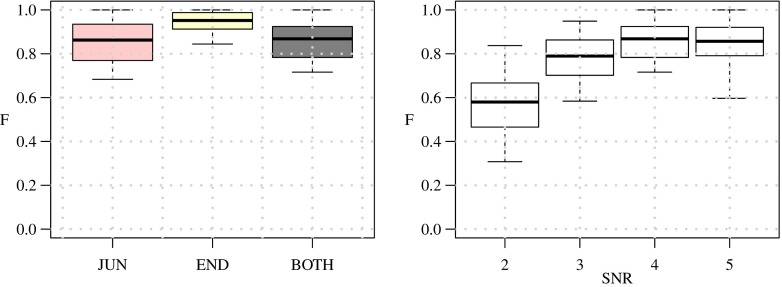
Performance of our method in detecting terminations and junctions in 30 simulated images of neurons. The distributions of the F_END_, F_JUN_, and F_BOTH_ values are shown as box plots for SNR=4 (*left panel*) and in addition the distribution of F_BOTH_ is shown for SNR=2, 3, 4, 5 (*right panel*)

**Fig. 16:**
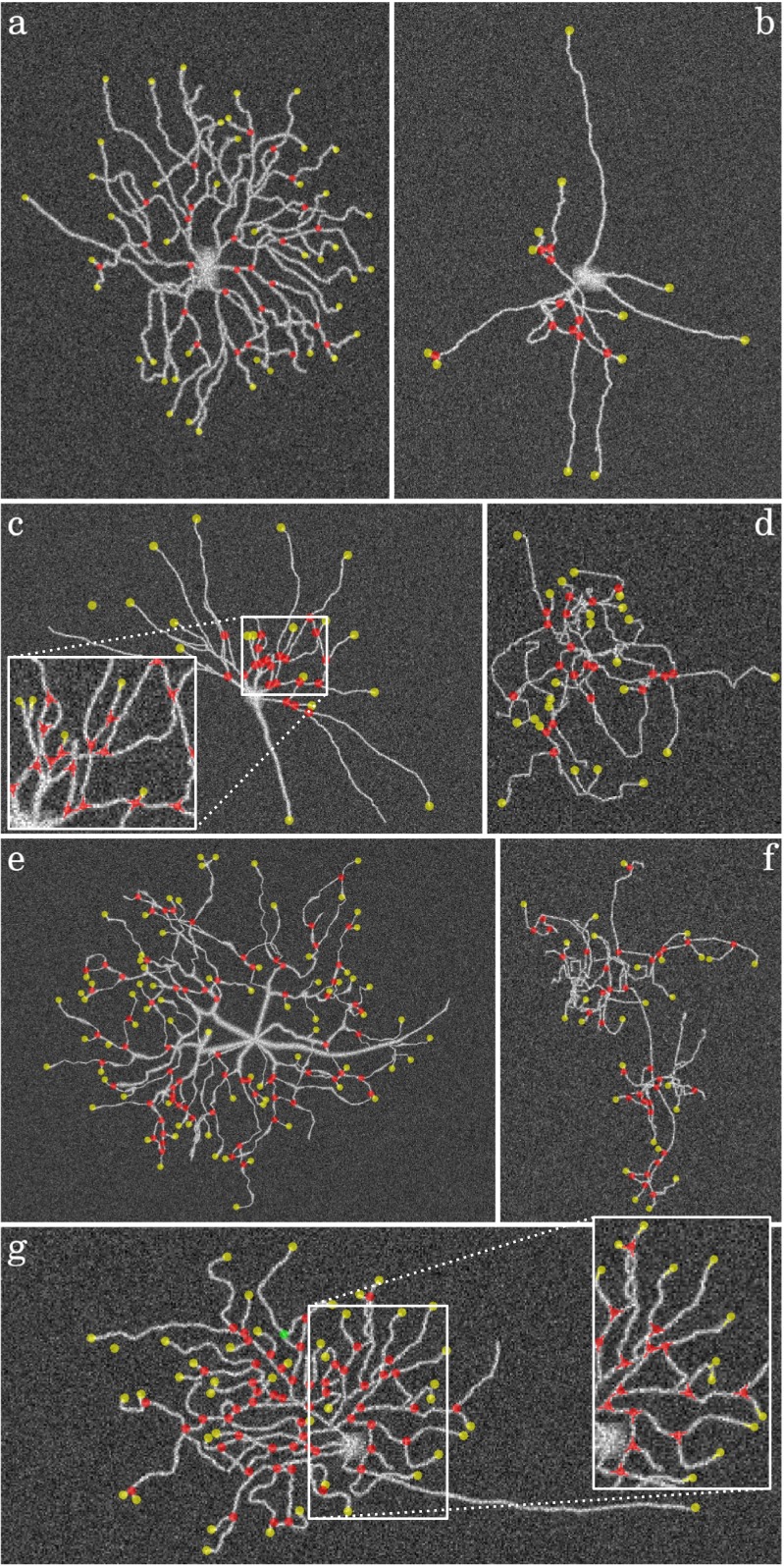
Example detection results in simulated neuron images at SNR=4. The images are contrast enhanced and show the detected terminations (*yellow circles*) and junctions (*red circles*) as overlays with fixed radius for better visibility. The value of F_BOTH_ in these examples is **a** 0.69, **b** 0.85, **c** 0.85, **d** 0.77, **e** 0.75, **f** 0.68, **g** 0.86

From the evaluation results (Fig. 15) we confirm the conclusion from the experiments on triplets that our method performs well for SNR≥4 and is somewhat better in detecting terminations than detecting junctions. For SNR=4 we find that the performance for junction detection is F_JUN_≈0.85 while for termination detection F_END_≈0.95. The higher performance for termination detection may be explained by the fact that the underlying image structure is usually less ambiguous (a single line-like structure surrounded by darker background) than in the case of junctions (multiple line-like structures that are possibly very close to each other). Example detection results are shown in Fig. 16.

### Evaluation on Real Neuron Images

**Fig. 17:**
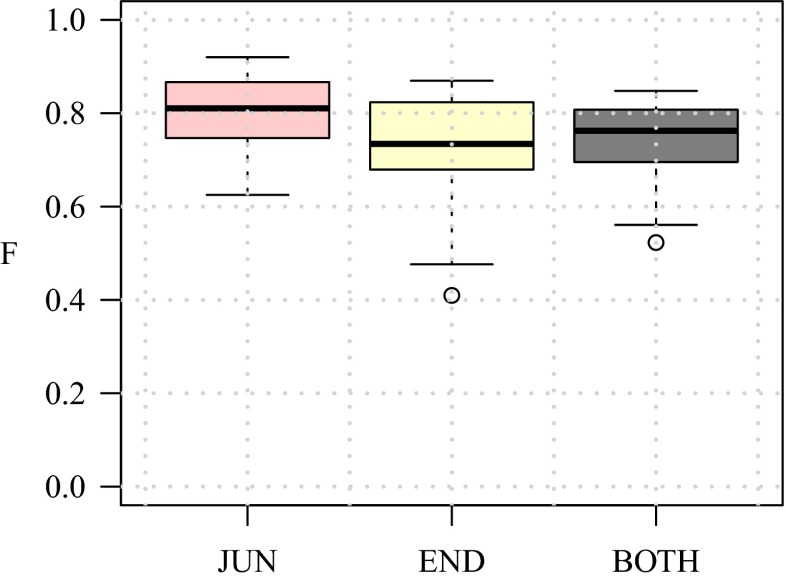
Performance of our method in detecting terminations and junctions in 30 real fluorescence microscopy images of neurons. The distributions of the F_END_, F_JUN_, and F_BOTH_ values for all images together are shown as box plots

**Fig. 18:**
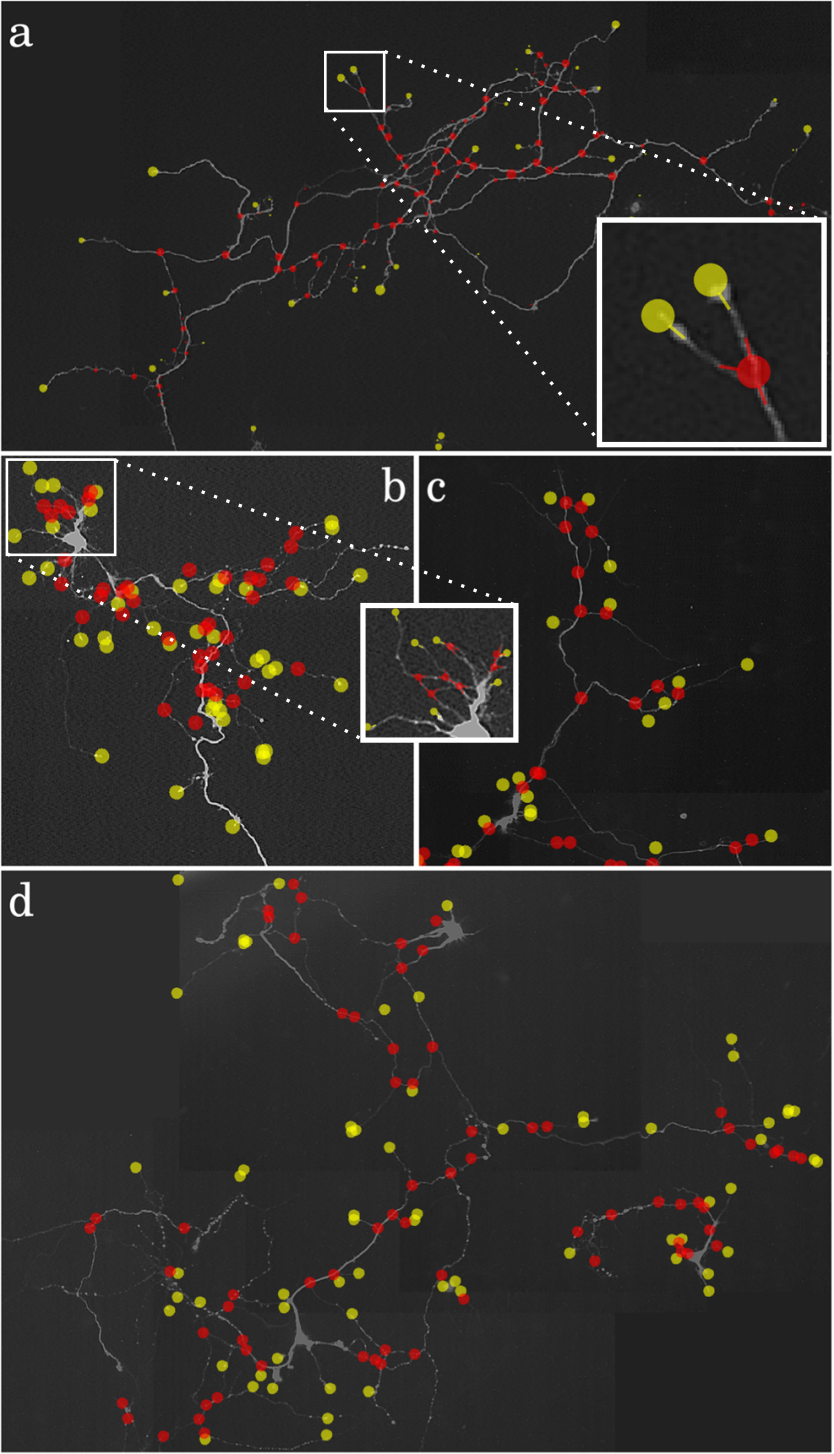
Example detection results for four real neuron images. The images show the detected terminations (*yellow circles*) and junctions (*red circles*) as overlays with fixed radius for better visibility. The value of F_BOTH_ in these examples is **a** 0.82, **b** 0.78, **c** 0.68, **d** 0.65

As the ultimate test case we also evaluated our method on real fluorescence microscopy images of neurons from a published study (Steiner et al. 2002). A total of 30 representative images were taken and expert manual annotations of the critical points were obtained to serve as the gold standard in this experiment. Needless to say, real images are generally more challenging than simulated images, as they contain more ambiguities due to labeling and imaging imperfections, and thus we expected our method to show lower performance. Since in this case we have no control over the SNR in the images we report the detection results of all images together. From the evaluation results (Fig. 17) we find that the median performance in detecting critical points is F_JUN_ = 0.81 for junctions and F_END_ = 0.73 for terminations while F_BOTH_ = 0.76. As expected, these numbers are lower than those of the simulated neuron images. Surprisingly, we observe that in the real images our method is better in detecting junctions than detecting terminations. A possible explanation for this could be that in the simulated images we used a constant intensity for the neuron branches, as a result of which terminations are as bright as junctions but much less ambiguous due to a clear background, while in the real images the terminations are usually much less clear due to labeling imperfections and the fact that the branch tips tend to be thinner and thus less bright than the junctions. This illustrates the limitations of the simulations. Example detection results are shown in Fig. 18.

### Comparison With Other Methods

Finally we sought to compare the performance of our Neuron Pinpointer (NP) method with other methods. Since we were not aware of other methods explicitly designed to detect and classify critical points in neuron images before reconstruction, we considered two existing software tools relevant in this context and we compared their implicit detection capabilities with our explicit method. If our method performs better, this would indicate that the existing methods may be improved by exploiting the output of our method.

The first tool, AnalyzeSkeleton (AS) (Arganda-Carreras et al. 2010), available from http://fiji.sc/AnalyzeSkeleton, is an ImageJ plugin for finding and counting all end-points and junctions in a skeleton image. To obtain skeleton images of our neuron images, we used the related skeletonization plugin available from the same developers, http://fiji.sc/Skeletonize3D, which is inspired by an advanced thinning algorithm (Lee et al. 1994). The input for the latter is a binary image obtained by segmentation based on smoothing (to reduce noise) and thresholding. For our experiments we considered a range of smoothing scales and manually selected thresholds as well as automatically determined thresholds using the following algorithms from ImageJ: Intermodes, Li, MaxEntropy, RenyiEntropy, Moments, Otsu, Triangle, and Yen. All of these were tried in combination with the AS method and the highest F-scores were used.

The second tool, All-Path-Prunning (APP2) (Xiao and Peng 2013), is a plugin for Vaa3D (Peng et al. 2010; Peng et al. 2014), available from http://www.vaa3d.org/. It was not designed specifically for a priori critical-point detection but for fully automatic neuron reconstruction. Nevertheless, in producing a tree representation of a neuron, the reconstruction algorithm must somehow identify the branch end-points and junctions, and for our experiments we can easily retrieve them from the SWC output files. In principle, any neuron reconstruction method is also implicitly a critical-point detection method, and we can quantify its performance by comparing the output tree nodes with the reference data. The interesting question is whether an explicit detector such as NP outperforms the implicit detection carried out in a tool such as APP2. We manually adjusted the user parameters of the tool to get optimal performance in our experiments.

**Fig. 19:**
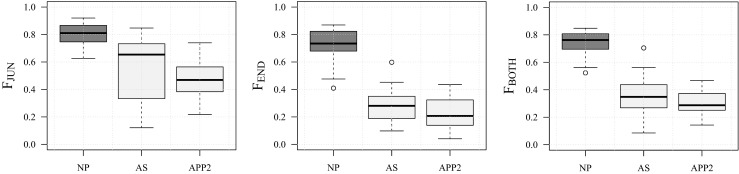
Critical-point detection performance of our method (NP) compared to two other methods (AS and APP2). The median values of F_JUN_ (*left plot*) are 0.81 (NP), 0.65 (AS), and 0.47 (APP2). The median values of F_END_ (*middle plot*) are 0.73 (NP), 0.28 (AS), and 0.21 (APP2). Finally, the median values of F_BOTH_ (*right plot*) are 0.76 (NP), 0.35 (AS), and 0.29 (APP2)

A comparison of the F-scores of NP, AS, and APP2 for the 30 real neuron images used in our experiments is presented in Fig. 19. From the plots we see that the detection rates of our NP method are substantially higher than those of AS and APP2. The difference is especially noticeable for the termination points. More specifically, the difference between F_END_ and F_JUN_ is relatively small for NP, but much larger for both AS and APP2. This indicates a clear advantage of using our explicit and integrated approach for detecting critical points, as accurate neuron reconstruction requires accurate detection of both junctions and terminations. However, with the current implementation, this advantage does come at a cost: timing of the three methods on a standard PC (with Intel Core i7-2630QM 2GHz CPU and 6 GB total RAM) revealed that with our images of 10^5^ to 10^6^ pixels in size, NP took about 40 seconds per image on average, while both AS and APP2 took only about 1.5 seconds per image. Fortunately, since virtually all the computation time of our method is spent in the directional filtering step, which is highly parallelizable, this cost can be reduced to any desired level by employing many-core hardware (such as GPUs).

## Conclusions

We have presented a novel method for solving the important problem of detecting and characterizing critical points in the tree-like structures in neuron microscopy images. Based on a directional filtering and feature extraction algorithm in combination with a two-stage fuzzy-logic based reasoning system, it provides an integrated framework for the simultaneous identification of both terminations and junctions. From the results of experiments on simulated as well as real fluorescence microscopy images of neurons, we conclude that our method achieves substantially higher detection rates than the rates that can be inferred from existing neuron reconstruction methods. This is true for both junction points and termination points, but especially for the latter, which are of key importance in obtaining faithful reconstructions. Altogether, the results suggest that our method may provide important clues to improve the performance of reconstruction methods.

Actual integration of our detection method with existing tracing methods was outside the scope of the present study, but we are currently in the process of developing a new neuron tracing method and, in that context, we aim to perform an extensive evaluation of the beneficial effects of the presented method also on existing tracing methods. For this purpose we also aim to extend our method to 3D, where the exact same workflow could be used, except that the angular profile analysis and the final critical-point determination step would involve two angles (azimuth and elevation) instead of one. Also, it would require mass parallelization of the image filtering step to keep the running times of the method acceptable, but this should be straightforward in view of the highly parallel nature of this step.

Although we focused on neuron analysis in this work, our method may also be potentially useful for other applications involving tree-like image structures, such as blood vessel or bronchial tree analysis, but this requires further exploration. For this purpose it may be helpful to increase the robustness of the detection method to larger branch diameter ratios than tested in this paper. This could be done, for example, by using multiscale filtering approaches, or by selective morphological thinning (or thickening).

## Information Sharing Statement

The software implementation of the presented method is available as an ImageJ (RRID:nif-0000-30467) plugin from https://bitbucket.org/miroslavradojevic/npinpoint.
